# Oncogenic Alterations in Histologically Negative Lymph Nodes Are Associated with Prognosis of Patients with Stage I Lung Adenocarcinoma

**DOI:** 10.3390/cancers14030824

**Published:** 2022-02-06

**Authors:** Yiping Tian, Qian Lai, Yuansi Zheng, Lisha Ying, Canming Wang, Jiaoyue Jin, Minran Huang, Yingxue Wu, Huizhang Li, Jianjun Zhang, Dan Su

**Affiliations:** 1 Department of Pathology, Cancer Hospital of the University of Chinese Academy of Sciences (Zhejiang Cancer Hospital), Hangzhou 310022, China; tianyp@zjcc.org.cn (Y.T.); laiqian@zjcc.org.cn (Q.L.); zhengyuansi2309@163.com (Y.Z.); wangcm@zjcc.org.cn (C.W.); jinjy@zjcc.org.cn (J.J.); huangmr@zjcc.org.cn (M.H.); 2 Institute of Basic Medicine and Cancer (IBMC), Chinese Academy of Sciences, Hangzhou 310022, China; yingls@zjcc.org.cn (L.Y.); yingxuewu1995@163.com (Y.W.); lihz@zjcc.org.cn (H.L.); 3 Department of Experimental Research Center, Cancer Hospital of the University of Chinese Academy of Sciences (Zhejiang Cancer Hospital), Hangzhou 310022, China; 4 Department of Cancer Prevention (DCP), Cancer Hospital of the University of Chinese Academy of Sciences (Zhejiang Cancer Hospital), Hangzhou 310022, China; 5 Department of Thoracic/Head and Neck Medical Oncology, University of Texas MD Anderson Cancer Center, Houston, TX 77030, USA; 6 Department of Genomic Medicine, University of Texas MD Anderson Cancer Center, Houston, TX 77030, USA

**Keywords:** oncogenic alteration, lymph node, stage I lung adenocarcinoma, prognosis, genomic analysis

## Abstract

**Simple Summary:**

Lymph nodes (LNs) metastasis is one of the most important factors affecting the outcome of non-small cell lung. The aim of this study is to explore whether presence of oncogenic alterations in histologically-negative lymph nodes (LNs) can be of prognostic significance in stage I lung adenocarcinoma (LUAD). We confirmed that presence of oncogenic alterations in regional LN may be associated with higher risks of postsurgical recurrence of Stage I LUAD, particularly for certain molecular subgroups. These results warranted future studies on larger cohort of NSCLC patients using more comprehensive cancer gene panels to establish the clinical impact of molecular LN occult metastasis for localized NSCLC and identify Stage I patients at high risks for recurrence for appropriate adjuvant therapy.

**Abstract:**

*Background*: Survival of patients with stage I non-small cell lung cancer (NSCLC) varies greatly. We sought to explore whether presence of oncogenic alterations in histologically-negative lymph nodes (LNs) can be of prognostic significance in stage I lung adenocarcinoma (LUAD). *Methods*: Genomic analysis of oncogenic alterations was applied to 123 stage I LUAD tumors. The same genomic variants identified in primary tumors were examined in corresponding histologically-negative LNs. *Results*: A total of 102 (82.9%) patients had at least one canonical oncogenic alteration detected in primary tumors, and 57 LNs from 12 patients (11.8%) were found to carry the identical oncogenic alterations detected in the corresponding primary tumor tissues, including *EGFR* mutations (six cases), *KRAS* mutations (three cases), *ALK* fusion (one case), *BRAF* mutation (one case) and *HER2* & *NRAS* co-mutations (one case). None of these LNs was found to have occult tumor cells by routine pathological assessment or immunohistochemistry staining using antibodies against pan-cytokeratins (AE1/AE3) and the epithelial marker Ber-EP4. The detection rate of oncogenenic alterations in LN was significantly higher in *RAS*-mutant tumors than *EGFR* mutant tumors (36.36% verse 7.41%, *p* = 0.017). Patients with oncogenic alterations in LN showed inferior disease-free survival (DFS, *p* = 0.025) and overall survival (OS, *p* = 0.027). Furthermore, patients with *RAS*-mutations detected in LN had the worst DFS and OS (*p* = 0.001). Among the 11 patients with *RAS* mutation in primary tumors, DFS and OS in the four patients with mutations detected in LN were significantly shorter than the remaining seven patients without mutations LN (DFS, *p =* 0.001, OS, *p =* 0.002). *Conclusions*: Genomic analysis has the potential to detect oncogenic alterations in regional LNs for localized LUAD and presence of oncogenic alterations in regional LN may be associated with inferior clinical outcome of stage I LUAD, particularly for certain molecular subgroups. ClinicalTrials.gov ID NCT04266691

## 1. Introduction

Lung cancer is the leading cause of cancer deaths worldwide. Lymph node (LN) metastasis is considered as one of the most important prognostic factors affecting the survival of localized non-small cell lung cancer (NSCLC). The 5-year survival is less than 50% for patients with LN positive NSCLC [1]. On the other hand, the prognosis varies greatly in patients without LN involvement [2]. One possible explanation are occult micrometastases (OM) in regional LNs undetected by routine pathological assessment [3,4], leading to inaccurate staging and suboptimal treatment. Improved LN staging is of importance for more accurate prognostication and to identify patients, who are at higher risks of postsurgical recurrence and may benefit from adjuvant therapy.

In the era of precision medicine, diagnosis and prognostication of cancer patients rely heavily on molecular biomarkers in addition to traditional pathology assessment. Over the past decade, great efforts have been made to improve methods to detect OMs at the molecular level by immunohistochemical (IHC) for epithelium-specific proteins or tumor associated proteins [5,6,7], reverse transcription-polymerase chain reaction (RT-PCR) of tumor associated mRNA [3,8] etc. However, since these markers were not specific to lung cancer, the accuracy of these assays has been in question. Moreover, whether OM in LNs is associated with higher risk of recurrence and poorer survival remains controversial, likely due to suboptimal markers and/or technologies [3,6,9,10].

Genomic aberrations in oncogenes such as epidermal growth factor receptor (*EGFR*) mutations and anaplastic lymphoma kinase (*ALK*) fusions have defined different molecular subtypes of NSCLC with unique cancer biology and response to matched tyrosine kinase inhibitors (TKI) [11]. As these oncogenic drivers are highly specific to certain molecular subtypes of NSCLCs, they could be potentially ideal biomarkers to detect lymph node molecular alterations. In the present study, we sought to employ genomic analysis of canonical oncogene alterations frequently identified in lung adenocarcinoma (LUAD) in histologically-negative LNs from stage I LUAD patients to detect molecular alterations and assess its prognostic value.

## 2. Patients and Methods

### 2.1. Patients

Patients with stage I LUAD who underwent surgical resection with curative intent between January 2009 and December 2015 in the Cancer Hospital of the University of Chinese Academy of Sciences in China (study registration number: NCT 04266691 at ClinicalTrial.Gov). All enrolled patients had mediastinal LN dissection and the treating surgeons decided the stations of LN sampling. Pathological diagnoses were independently confirmed by two pathologists. Only patients with pathological stage I disease with tumor  ≤4 cm (T1–T2aN0M0) based on the 8th Edition of the American Joint Committee on Cancer (AJCC) Staging Manual were included in this study [12]. Patients who received neoadjuvant chemotherapy, had R1 resection or inadequate tumor specimens were excluded from subsequent genomic analysis. All patients were followed up until death or censored since the date from their primary surgery. The survival data was locked on 26 June 2019. This study was approved by the institutional review board of the Cancer Hospital of the University of Chinese Academy of Sciences (No. IRB-2021-387).

### 2.2. Specimen Processing and DNA/RNA Extraction

The formalin-fixed paraffin-embedded (FFPE) tumor tissues from each patient was collected and subjected to DNA/RNA co-extraction using DNA and RNA Extraction Kits (Amoy Diagnostics, Xiamen, China). If gene alterations were identified in the primary LUAD tumors, the corresponding LNs were collected and DNA/RNA was subsequently extracted separately. Genomic analysis for LNs were performed on 10-μm-thick FFPE tissues using tissue scrolls. AmoyDx FFPE DNA Kit (Amoy Diagnostics) or AmoyDx FFPE RNA Kit (Amoy Diagnostics) was used for DNA or RNA extraction from LNs, respectively.

### 2.3. Detection of Oncogenic Gene Alterations in Lung Cancer Specimens

Oncogenic alterations of *EGFR, ALK, ROS1, RET, KRAS, BRAF, HER2, NRAS,* and *PIK3CA* were detected by fluorescence quantitative PCR in tumor samples using Stratagene Mx3000P™ and AmoyDx Multi-Gene Mutations Detection Kit (Amoy Diagnostics, Xiamen, China). Fusions of *ALK*, *ROS1* and *RET* genes were identified by mRNA-based methods using specific primers and fluorescent probes in a one-step RT-PCR [13] and point mutations in *EGFR*, *KRAS*, *BRAF*, *NRAS*, *HER2*, and *PIK3CA* genes were identified using the ADx-amplification refractory mutation system (ARMS) as previously described [14]. Only the canonical oncogenic mutations such as *EGFR* L858R/19Del/T790M, *KRAS* G12C/A/V/R, *BRAF* V600E, *NRAS* G12C/R/V/A, *PIK3CA* E545K, *HER2* 20ins etc. were included in the current study. This sensitive ARMs-qPCR assay can detect mutations present in as low as 1% of cell population [15]. The results were confirmed according to the manufacturer’s guidelines. 

### 2.4. Detection of Oncogenic Gene Alterations in Lymph Nodes

If genetic alterations were found in the primary lung cancer tissues, the corresponding LNs were examined for these same variants. A total of 1418 LNs from different LN stations were classified based on 8th Edition of the AJCC staging and all the patients did mediastinal lymph node dissection [12]. N1 LNs included ipsilateral peribronchial and/or ipsilateral hilar LNs and intrapulmonary nodes, while N2 LNs included ipsilateral mediastinal and/or subcarinal LNs. LNs of the same LN station were pooled for DNA/RNA extraction and sequencing. Skip metastasis in N2 LNs was defined as driver gene mutations detected in N2 but not in N1 LNs. All histologically negative LNs were examined by two pathologists independently prior to DNA/RNA extraction.

### 2.5. Immunohistochemical (IHC) Detection of Occult Lymph Node Metastases

IHC assay was performed to detect micrometastases using antibodies against pan-cytokeratin (AE1/AE3, dilution 1:100, ZhongShan JinQiao, Beijing, China) and the epithelial marker Ber-EP4 (dilution 1:25, Maixin, Fujian, China) [3,16] in LNs with mutations detected by PCR assay. FFPE tissue blocks were cut into 4-μm-thick sections and attached to a positive-charged glass slide. IHC staining of AE1/AE3 and Ber-EP4 were carried out with a Bond-III automatic IHC staining device (Leica Biosystems, Wetzlar, Germany) as previously described [17]. Small tumor deposits or solitary tumor cells were defined as micrometastases or occult metastases.

### 2.6. Statistical Analysis

Statistical analysis was performed using SPSS statistics version 22.0 (IBM Corp., Armonk, NY, USA). The categorical variables were described as number (percentage) and the continuous variables were described as means and standard deviations (SD). Pearson Chi-square test or Fisher’s exact test was used to compare between categorical variables, as appropriate. Kaplan-Meier (K-M) analysis was used and differences between the groups were evaluated by the log-rank test. The overall survival (OS) was defined as the time from the date of the first surgery to the date of death from any cause or the date of the last follow-up. Disease-free survival (DFS) was considered as the time from the date of the initial surgery until the date of diagnosis of the initial recurrence or death from any cause. A two-sided *p*-value < 0.05 was considered statistically significant.

## 3. Results

### 3.1. Patients’ Characteristics

**Figure 1:**
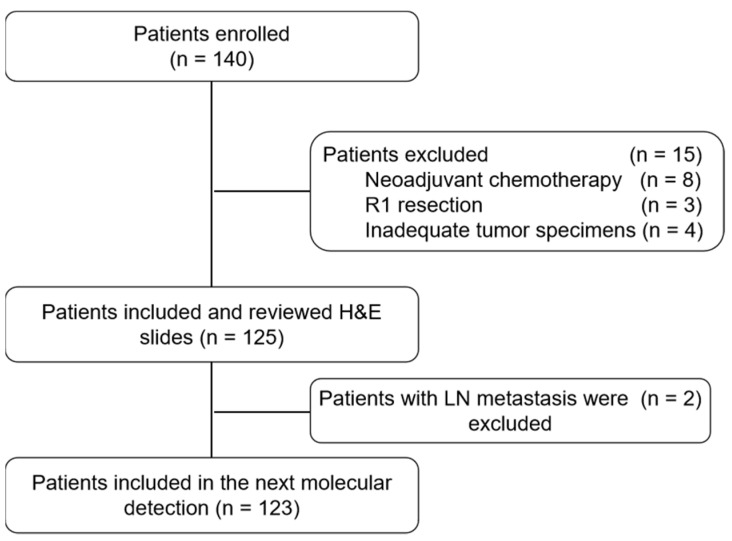
Study scheme. A total of 140 patients were initially quired and 17 patients were excluded because of receiving neoadjuvant chemotherapy (n = 8), R1 resection (n = 3), inadequate tumor specimens (n = 4) or identification of LN metastasis at the time of FFPE block sectioning. Finally, the remaining 123 eligible patients were subjected to subsequent analyses.

A total of 140 patients with stage I LUAD who underwent surgical resection with curative intent between January 2009 and December 2015 and had final pathology staging of pN0 were enrolled (NCT 04266691). Fifteen patients were excluded including eight patients who received neoadjuvant chemotherapy, three patients with R1 resection and four patients with inadequate tumor specimens (Figure 1). The H&E slides of all primary tumors and LNs from these 125 patients were independently reviewed by two experienced pathologists to confirm the diagnosis. All LNs were re-cut and the first as well as the last recut slides were evaluated to rule out any metastatic involvement. LN metastasis was identified in two of the 125 (1.6%) patients after meticulous review. A small locus of metastasis (2.2 mm) was missed in the original paraffin section in one patient and for the other patient, metastasis was not present in the original section, but a 1.6 mm locus of metastasis was found after re-sectioning. These two patients were excluded from the subsequent genomic analyses (Figure 1). 

The remaining 123 cases were subjected to genomic analysis. There were 78 (63.4%) male and 45 (36.6%) female patients, with a mean age of 61 (ranging from 44 to 77 years old). Never smokers constituted 56.1% (n = 69) of the cohort and 41 (33.3%) patients had a family history of lung cancer (Table 1). All patients had peripheral LUAD. 113 (91.9%) patients received lobectomies and 10 (8.1%) patients received segmentectomies. All patients had mediastinal lymph node dissection. Among the 123 patients, seven were found to have only N1 LNs but no N2 LN tissues (fibrous tissues instead) in the final pathology despite mediastinal lymph node dissection was performed. The mean number of resected lymph nodes was 14.5 ± 7.4 (range 1 to 38). With a median follow up of 48.7 months, 24 (19.5%) patients have recurred and 12 of these 24 patients expired, while all 99 (80.5%) patients without recurrence were alive at the date of data lock. The median disease-free survival (DFS) was 47.64 months (ranging from 1 to 113 months) and the median overall survival (OS) was 48.69 months (ranging from 12 to 113 months). Age, sex, smoking status, tumor size or lympho-vascular invasion was not found to associate with the patients’ outcome, which may be due to small sample size of the current cohort [18,19,20,21].

### 3.2. Oncogenic Alternations in Primary Lung Adenocarcinoma Specimens

As shown in Table 1, at least one oncogenic alteration was detected in the primary lung cancer tissues from 102 of the 123 patients (82.93%) including *EGFR* mutations in 81 patients (65.85%), *KRAS* mutations in nine patients (7.32%), *ALK* fusions in three patients (2.44%), *NRAS* mutations in one patient (0.81%), *HER2* mutations in two patients (1.63%), *RET* fusions in two patients (1.63%), *ROS1* fusion in one patient (0.81%), *BRAF* mutation in one patient (0.81%), *PIK3CA* & *EGFR* co-mutations in one patient (0.81%) and *HER2* & *NRAS* co-mutations in one patient (0.81%) (Table 2). Presence of driver mutations was associated with never smoker (Table 1).

### 3.3. Oncogenic Alternations Were Present in Histologically Negative Lymph Nodes

**Figure 2:**
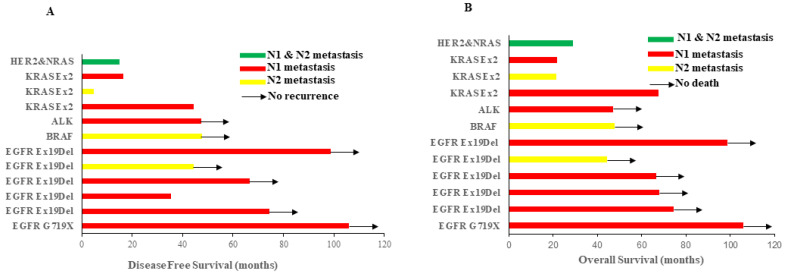
Disease-free survival (**A**) and overall survival (**B**) of patients with LN molecular alterations of different oncogenic mutations. Different colors represent those molecular alterations in different LN stations. Arrows indicate that patients had no recurrence or death.

If genetic alterations were identified in the primary lung cancer tissues, the LNs from the same patients were sequenced for the same genes. A total of 1418 morphologically negative LNs from 102 patients with oncogenic alterations identified in primary tumors were subjected to genomic analysis of same variants in their corresponding primary tumors. Totally, 57 LNs from 12 of the 102 patients (11.8%) were found to carry the identical oncogenic alterations detected in the corresponding primary NSCLC tumor tissues. Among 12 cases, six were *EGFR* mutations, three were *KRAS* mutations, one was an *ALK* fusion, one was a *BRAF* mutation and one was *HER2* & *NRAS* co-mutations (Table 3). To exclude occult tumor cell metastasis, all of these positive LNs were analyzed by IHC for pan-cytokeratin (AE1/AE3) and Ber-EP4 and none of these LNs had cancer cells detected (Figure S1). Among these 12 patients, eight patients had N1 LN-only oncogenic alternations (five with LN*^EGFR^* ^Mutation^, two with LN*^KRAS^* ^Mutation^, one with LN*^ALK^* ^Fusion^), three patients had N2 LN-only oncogenic alternations (one with LN*^EGFR^* ^Mutation^, one with LN*^KRAS^* ^Mutation^, one with LN*^BRAF^* ^Mutation^) and one patient was found to have oncogenic alternations in both N1 and N2 LNs (LN*^HER2^*^/*NRAS* co-Mutation^ in N1 while LN*^NRAS^* ^Mutation^ in N2, Figure 2 and Table 3). The rate of LN oncogenic alternations in patients with *RAS* (*NRAS* or *KRAS* or *NRAS/HER2*) was significantly higher than those with *EGFR* mutation (4/11 verse 6/81, *p* = 0.017, Table 4). 

### 3.4. Oncogenic Alternations in LN Impacts Survival

With the small sample size fully acknowledged, we next sought to investigate whether the presence of oncogenic alternations in LN impacts survival of histologically LN-negative stage I LUAD. The median age of these 12 patients was 56.4 years old with the median DFS of 51.9 months and median OS of 70 months (Figure 2 and Table 3). Among these 12 patients, all three patients with LN*^KRAS^* ^Mutation^ recurred at 45.6, 4.9, 16.4 months and expired at 67.6, 21.5 and 21.9 months post-surgery, respectively. The patient with LN*^HER2^* ^& *NRAS* co*-*Mutations^ also recurred 15 months and expired 29 months post-surgery. On the other hand, only one of the six patients with LN*^EGFR^* ^Mutation^ recurred 6 months post-surgery and was still alive while the other five patients were all alive without recurrence at the time of data lock, with a median DFS of 70.6 months and median OS of 71.2 months. In addition, the patient with LN*^ALK^* ^fusion^ and the patient with LN*^BRAF^* ^Mutation^ did not recur with OS of 47.31 months and 47.74 months, respectively. 

**Figure 3:**
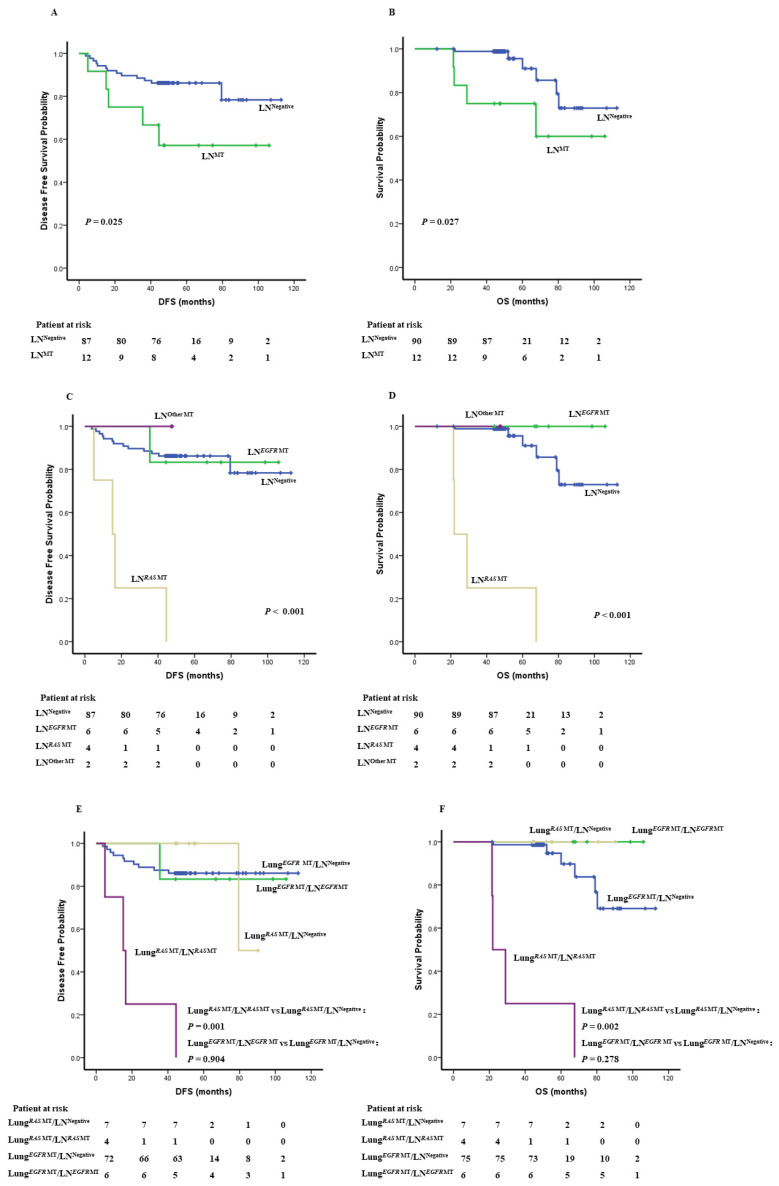
Survival of 102 histologically stage I LUAD with or without molecular alterations in LN. Comparison of DFS (**A**) and OS (**B**) between patients with molecular alterations in LN (LN^MT^, green line) versus those without molecular alterations in LN (LN^Negative^, blue line). Comparison of DFS (**C**) and OS (**D**) of patients carrying *EGFR* molecular alterations in LN (LN*^EGFR^* ^MT^, green line), *RAS* molecular alterations in LN (LN*^RAS^* ^MT^*,* yellow line), other molecular alterations in LN (LN*^Other^* ^MT^ purple line) and patients without molecular alterations in LN (LN^Negative^, blue line). Comparison of DFS (**E**) and OS (**F**) of *EGFR*-mutant patients with molecular alterations in LN (Lung*^EGFR^* ^MT^LN*^EGFR^* ^MT^, green line), *EGFR*-mutant patients without molecular alterations in LN (Lung*^EGFR^* ^MT^LN ^Negative^, blue line), *RAS-*mutant patients with molecular alterations in LN (Lung*^RAS^* ^MT^LN*^RAS^* ^MT^*,* purple line), and *RAS-*mutant patients without molecular alterations in LN (Lung*^RAS^* ^MT^LN ^Negative^, yellow line). *p* was determined with the log-rank test.

Among the 102 patients with oncogenic alternations detected in primary tumor tissues, the median DFS or OS has not been reached, but the 12 patients with oncogenic alternations in LN had shorter DFS and OS (DFS: *p* = 0.025, OS: *p* = 0.027, Figure 3A,B). Of particular interest, patients with *RAS* mutations identified in LN (including *KRAS* mutation and *HER2* & *NRAS* co-mutations) showed the worst DFS and OS (*p* < 0.001, Figure 3C,D) among these 102 patients, suggesting *RAS* mutations in LN may be a poor prognostic factor for histologically stage I LUAD. 

Furthermore, among the 11 patients with *RAS* mutations in primary lung cancer tissues, DFS and OS in the four patients with *RAS* mutations detected in LN were significantly shorter than the remaining seven patients without mutations in LN (DFS: 15.1 months versus 90 months, *p* = 0.001; OS: 21.9 months versus 90 months, *p* = 0.002). On the other hand, among the 81 patients with *EGFR* mutations detected in primary lung cancer tissues, the median DFS or OS has not been reached and the survival of the six patients with mutations detected in LN was not different from their counterparts without mutations in LN (DFS: *p* = 0.904; OS: *p* = 0.278, Figure 3E,F).

## 4. Discussion

Lymph node metastasis is one of the most important prognostic factors for many localized malignancies, including LUAD [22,23] and routine histologic assessment may not be always satisfactory. Serial sectioning of sentinel LN has been demonstrated efficacy to detect small metastases in melanoma [24]. We were able to identify small LN metastasis from two patients that were missed by routine postsurgical pathologic assessment in the current study. However, serial LN sectioning and histological assessment of LNs is time-consuming and subjective to human errors. As such, molecular OM have been scrutinized. For example, it was shown that dissemination of lung cancer cells to regional LN and distant organs can be detected by immunohistochemical (IHC) using monoclonal antibodies against epithelium-specific proteins or tumor-specific biomarkers [5,6,7]. Reverse transcription-polymerase chain reaction (RT-PCR) of tumor associated mRNA such as carcinoma embryonic antigen (CEA) mRNA [3] and mucin type 1 (MUC1) mRNAs [8] was also demonstrated having the potential to identify a small number of tumor cells in histologically negative LN from NSCLC patients. However, none of these markers is specific to lung cancers and RNA/protein expression of these genes may vary significantly in different cancer cells (spatial heterogeneity) [25,26] and change during disease course (temporal heterogeneity) [27,28]. These limitations have precluded the prognostic values of these assays. Genomic alterations of cancer genes are ideal markers because: (1) cancer gene alterations are often specific to cancers; (2) the majority of these cancer gene mutations are clonal thus are present in all tumor cells [29] including LN metastasis [30]; (3) the technologies for genomic analysis are mature and reproducible. 

In the current study, we sought to assess the feasibility of genomic analysis of oncogenes to detect molecular OM in histologically negative LNs in stage I LUAD. Oncogenic alternations were identified from 12 patients, accounting for 11.8% of patients with oncogenic alternations detected in primary tumors, which further confirmed by IHC that showed no isolated tumor cells. This incidence of 11.8% was likely underestimated since only nine oncogenes were analyzed. As such, genomic alternations in regional LNs may be detected in a much larger proportion of stage I LUAD patients if additional commonly mutated cancer genes were included. These findings may be of significant impact given the high incidence of lung cancers.

Interestingly, the detection rate of oncogenic alterations in LNs was significantly higher in *RAS-*mutant (*NRAS* or *KRAS* or *NRAS/HER2*) patients than *EGFR*-mutant patients in our cohort (Table 4). Furthermore, patients with *RAS* mutations detected in LNs had significant shorter survival (Figure 3C,D). Lung cancer is a heterogeneous disease. In addition to histology and staging, cancer gene mutations define cancer biologic and clinical features including patterns of local and distant spread. For example, Liu et al. reported that *ROS1* rearrangement, *RET* mutation and *ALK* rearrangement had higher risks of LN metastasis than other genotypes [31]. Our results suggested that *RAS*-mutant lung cancers may be at higher risks having molecular OM in regional LN.

The next important question is whether LN OM defined by the presence of oncogenic alterations can inform adjuvant therapies. Adjuvant chemotherapy only provides minimal if any survival benefit for patients with stage I NSCLC [32]. As such adjuvant chemotherapy is not recommended for patients with stage I NSCLC without other high-risk features although a considerable proportion of stage I lung cancer patients still recur [32,33]. As the goal of adjuvant therapy is to eliminate OM (stages and other high-risk features are surrogates for OM), detecting actual OM using molecular assays such as liquid biopsy and molecular profiling of histologically negative LN can serve as better surrogates to identify high-risk patients with OM, who may benefit from adjuvant therapy. However, routinely profiling all histologically LNs from all patients can put a large financial burden to patients and/or medical system. As such, these assays should be applied selectively to patients who may benefit from certain adjuvant therapies. Importantly, with the data emerging to support adjuvant targeted therapy in patients with targetable mutations [34] and immune therapy in patients without [35], molecular profiling has been gradually adapted to resected NSCLCs tumors. While it is still controversial whether all stage I lung cancer patients with targetable molecular changes would benefit from adjuvant TKI, patients with confirmed molecular OM in LNs are reasonable candidates for adjuvant targeted therapies. Therefore, a potentially cost-effective approach would be to test LNs from patients with stage I lung cancer that had mutations detected in primary tumors. If mutations are detected in LNs, these patients may be at higher risk of recurrence and therefore can be considered for adjuvant targeted therapy, chemotherapy and/or immunotherapy. Furthermore, pooling DNA from multiple LNs and using targeted panel based on the mutations detected in the primary tumors could be considered to further reduce the cost.

One caveat of studies on oncogenic alterations in regional LN, including the current study is that the definition of molecular occult metastasis is still controversial. For example, in breast cancers, even isolated tumor cells (ITC; or ≤0.2 mm) in LNs detected by HE/IHC or positive molecular findings by RT-PCR without evidence for metastasis do not correlate with recurrence or survival and are therefore not defined as metastasis-positive cells in the TNM classification [36,37,38]. In our study, the oncogenic alterations identical to those in primary LUAD tumors were detected in regional LNs indicating these genomic alterations were from the same cancer cells. However, whether these alterations were from micro-metastatic cancer cells in LN or cell free tumor DNA transported by lymph fluid [39,40,41] or phagocytic immune cells of the regional lymph nodes [42,43] is unclear. Nevertheless, even with only 12 patients with oncogenic alterations detected in LN, we observed a trend that presence of oncogenic mutations in regional LN may be associated with inferior clinical outcome, particularly in patients with *RAS*-mutant LUADs. 

To the best of our knowledge, this is the first study to identify oncogenic mutations in histologically-negative LNs on a large cohort of stage I LUAD patients. As a proof-of-concept, our results demonstrated that is feasible to detect cancer gene alterations in regional LN of localized LUAD by genomic analysis and suggested that the presence of oncogenic alterations in regional LN may be associated with higher risks of postsurgical recurrence of stage I LUAD, particularly for certain molecular subgroups. These intriguing results warrant future studies on a larger cohort of NSCLC patients using more comprehensive cancer gene panels to establish the clinical impact of molecular LN occult metastasis for localized NSCLC and identify stage I patients at high risks for recurrence for appropriate adjuvant therapy.

## 5. Conclusions

Genomic analysis has the potential to detect oncogenic alterations in regional LNs for stage I LUAD and presence of oncogenic alterations in regional LN may be associated with inferior clinical outcome of stage I LUAD, particularly for certain molecular subgroups. 

## Figures and Tables

**Table 1:** Clinical characteristics of 123 stage I LUAD with or without oncogenic alterations.

Characteristic		Total (N = 123)	Without Genetic Alterations in Tumor (N = 21)	With Genetic Alterations in Tumor (N = 102)	*p*-Value
Age	≤60	55 (44.7%)	12 (21.8%)	43 (78.2%)	
	>60	68 (55.3%)	9 (13.2%)	59 (86.6%)	0.208
Sex	Male	78 (63.4%)	16 (20.5%)	62 (79.5%)	
	Female	45 (36.6%)	5 (11.1%)	40 (88.9%)	0.182
Smoke	No	69 (56.1%)	6 (8.7%)	63 (91.3%)	
	Yes	54 (43.9%)	15 (27.8%)	39 (72.2%)	0.005
Family history	No	82 (66.7%)	11 (13.4%)	71 (86.6%)	
	Yes	41 (33.3%)	10 (24.4%)	31 (75.6%)	0.127
Tumor Size	≤3 cm	107 (87.0%)	18 (16.8%)	89 (83.2%)	
	>3 cm	16 (13.0%)	3 (18.8%)	13 (81.3%)	0.736
Surgery type	Lobectomies	113 (91.9%)	18 (15.9%)	95 (84.1%)	
	Segmentectomies	10 (8.1%)	3 (30.0%)	7 (70.0%)	0.372

*p* was calculated by Chi-square test or Fisher’s exact test.

**Table 2:** Oncogenic gene alterations status in primary lung cancer tissues from 123 patients.

Gene Type	Mutations in Primary Lung Cancer Tissues	
	No.	%
Wild Type	21	17.07
*EGFR* mutation	81	65.85
*KRAS* mutation	9	7.32
*NRAS* mutation	1	0.81
*HER2* mutation	2	1.63
*ALK* fusion	3	2.44
*RET* fusion	2	1.63
*ROS1* fusion	1	0.81
*BRAF* mutation	1	0.81
*PIK3CA*&*EGFR* mutations	1	0.81
*HER2*&*NRAS* mutations	1	0.81

**Table 3:** Clinical characteristics in 12 patients with lymph node molecular alterations.

Molecular Alteration Type	Number (Percentage)	Lymph Node Station	Average Age (Year)
Total	12		56.4
*EGFR* mutation	6 (50%)	N1; N2	63.5
*ALK* fusion	1 (8.3%)	N1	58
*KRAS* mutation	3 (25%)	N1; N2	59.6
*Her2*&*NRAS* mutaions	1 (8.3%)	N1 & N2	51
*BRAF* mutation	1 (8.3%)	N2	71

**Table 4:** Rate of LN molecular alterations in patients with *EGFR* or *RAS* mutations.

	No LN Molecular Alterations	LN Molecular Alterations	*p*
*EGFR* mutation	75 (92.59%)	6 (7.41%)	0.017
*RAS* mutation	7 (63.64%)	4 (36.36%)	

*p* was calculated by Fisher’s exact test.

## Data Availability

The data presented in this study are available on request from the corresponding author.

## Supplementary Materials

The following supporting information can be downloaded at: https://www.mdpi.com/2072-6694/14/3/824/s1, Figure S1: The expression of AE1/AE3 and BerEP4 in primary cancer tissue and corresponding lymph node with molecular alteration.

## References

[1] Benlloch S., Galbis-Caravajal J.M., Alenda C., Peiro F.M., Sanchez-Ronco M., Rodriguez-Paniagua J.M., Baschwitz B., Rojas E., Massuti B. (2009). Expression of molecular markers in mediastinal nodes from resected stage I non-small-cell lung cancer (NSCLC): Prognostic impact and potential role as markers of occult micrometastases. Ann. Oncol..

[2] Howington J.A., Blum M.G., Chang A.C., Balekian A.A., Murthy S.C. (2013). Treatment of Stage I and II Non-Small Cell Lung Cancer: Diagnosis and Management of Lung Cancer, 3rd ed: American College of Chest Physicians evidence-based clinical practice guidelines. Chest.

[3] Martin L.W., D–Cunha J., Wang X., Herzan D., Gu L., Abraham N., Demmy T.L., Detterbeck F.C., Groth S.S., Harpole D.H. (2016). Detection of Occult Micrometastases in Patients with Clinical Stage I Non-Small-Cell Lung Cancer: A Prospective Analysis of Mature Results of CALGB 9761 (Alliance). J. Clin. Oncol..

[4] Erhunmwunsee L., D–Amico T.A. (2008). Detection of occult N2 disease with molecular techniques. Thorac Surg Clin..

[5] Kubuschok B., Passlick B., Izbicki J.R., Thetter O., Pantel K. (1999). Disseminated tumor cells in lymph nodes as a determinant for survival in surgically resected non-small-cell lung cancer. J. Clin. Oncol..

[6] Gu C.D., Osaki T., Oyama T., Inoue M., Kodate M., Dobashi K., Oka T., Yasumoto K. (2002). Detection of micrometastatic tumor cells in pN0 lymph nodes of patients with completely resected nonsmall cell lung cancer: Impact on recurrence and Survival. Ann. Surg..

[7] Ohgami A., Mitsudomi T., Sugio K., Tsuda T., Oyama T., Nishida K., Osaki T., Yasumoto K. (1997). Micrometastatic tumor cells in the bone marrow of patients with non-small cell lung cancer. Annals Thorac. Surg..

[8] Saintigny P., Coulon S., Kambouchner M., Ricci S., Martinot E., Danel C., Breau J.L., Bernaudin J.F. (2005). Real-time RT-PCR detection of CK19, CK7 and MUC1 mRNA for diagnosis of lymph node micrometastases in non small cell lung carcinoma. Int. J. Cancer.

[9] Dobashi K., Sugio K., Osaki T., Oka T., Yasumoto K. (1997). Micrometastatic P53-positive cells in the lymph nodes of non-small-cell lung cancer: Prognostic significance. J. Thorac. Cardiovasc. Surg..

[10] Nicholson A.G., Graham A.N., Pezzella F., Agneta G., Goldstraw P., Pastorino U. (1997). Does the use of immunohistochemistry to identify micrometastases provide useful information in the staging of node-negative non-small cell lung carcinomas?. Lung Cancer.

[11] Recondo G., Facchinetti F., Olaussen K.A., Besse B., Friboulet L. (2018). Making the first move in EGFR-driven or ALK-driven NSCLC: First-generation or next-generation TKI?. Nat. Rev. Clin. Oncol..

[12] Detterbeck F.C., Boffa D.J., Kim A.W., Tanoue L.T. (2017). The Eighth Edition Lung Cancer Stage Classification. Chest.

[13] Bruno R., Giordano M., Giannini R., Ali G., Puppo G., Ribechini A., Chella A., Fontanini G. (2016). Aberrant expression of anaplastic lymphoma kinase in lung adenocarcinoma: Analysis of circulating free tumor RNA using one-step reverse transcription-polymerase chain reaction. Mol. Med. Rep..

[14] Xu H., Baidoo A.A.H., Su S., Ye J., Chen C., Xie Y., Bertolaccini L., Ismail M., Ricciuti B., Ng C.S.H. (2019). A comparison of EGFR mutation status in tissue and plasma cell-free DNA detected by ADx-ARMS in advanced lung adenocarcinoma patients. Transl. Lung Cancer Res..

[15] Shaozhang Z., Ming Z., Haiyan P., Aiping Z., Qitao Y., Xiangqun S. (2014). Comparison of ARMS and direct sequencing for detection of EGFR mutation and prediction of EGFR-TKI efficacy between surgery and biopsy tumor tissues in NSCLC patients. Med. Oncol..

[16] Herpel E., Muley T., Schneider T., Palm E., Kieslich de Hol D., Warth A., Meister M., Storz K., Schnabel P.A., Schirmacher P. (2010). A pragmatic approach to the diagnosis of nodal micrometastases in early stage non-small cell lung cancer. J. Thorac. Oncol..

[17] Tian Y., Sun X., Cheng G., Ji E., Yang S., Feng J., Zheng L. (2021). The association of CMTM6 expression with prognosis and PD-L1 expression in triple-negative breast cancer. Ann. Transl. Med..

[18] Detterbeck F.C. (2011). Pushing forward into the darkness, leaping, and landing securely: Prognostication and adjuvant chemotherapy for lung cancer. Chest.

[19] Zhang Y., Sun Y., Xiang J., Zhang Y., Hu H., Chen H. (2014). A clinicopathologic prediction model for postoperative recurrence in stage Ia non-small cell lung cancer. J. Thorac. Cardiovasc. Surg..

[20] Ou S.H., Zell J.A., Ziogas A., Anton-Culver H. (2007). Prognostic factors for survival of stage I nonsmall cell lung cancer patients: A population-based analysis of 19,702 stage I patients in the California Cancer Registry from 1989 to 2003. Cancer.

[21] Shimada Y., Saji H., Yoshida K., Kakihana M., Honda H., Nomura M., Usuda J., Kajiwara N., Ohira T., Ikeda N. (2013). Prognostic factors and the significance of treatment after recurrence in completely resected stage I non-small cell lung cancer. Chest.

[22] Qiu C., Dong W., Su B., Liu Q., Du J. (2013). The prognostic value of ratio-based lymph node staging in resected non-small-cell lung cancer. J. Thorac. Oncol..

[23] Yang J., Peng A., Wang B., Gusdon A.M., Sun X., Jiang G., Zhang P. (2019). The prognostic impact of lymph node metastasis in patients with non-small cell lung cancer and distant organ metastasis. Clin. Exp. Metastasis.

[24] Lobo A.Z., Tanabe K.K., Luo S., Muzikansky A., Sober A.J., Tsao H., Cosimi A.B., Duncan L.M. (2012). The distribution of microscopic melanoma metastases in sentinel lymph nodes: Implications for pathology protocols. Am. J. Surg Pathol..

[25] Lee W.C., Diao L., Wang J., Zhang J., Roarty E.B., Varghese S., Chow C.W., Fujimoto J., Behrens C., Cascone T. (2018). Multiregion gene expression profiling reveals heterogeneity in molecular subtypes and immunotherapy response signatures in lung cancer. Mod. Pathol..

[26] Herbst R.S., Soria J.C., Kowanetz M., Fine G.D., Hamid O., Gordon M.S., Sosman J.A., McDermott D.F., Powderly J.D., Gettinger S.N. (2014). Predictive correlates of response to the anti-PD-L1 antibody MPDL3280A in cancer patients. Nature.

[27] Mansfield A.S., Aubry M.C., Moser J.C., Harrington S.M., Dronca R.S., Park S.S., Dong H. (2016). Temporal and spatial discordance of programmed cell death-ligand 1 expression and lymphocyte tumor infiltration between paired primary lesions and brain metastases in lung cancer. Ann. Oncol..

[28] Aramaki N., Ishii G., Yamada E., Morise M., Aokage K., Kojima M., Hishida T., Yoshida J., Ikeda N., Tsuboi M. (2016). Drastic morphological and molecular differences between lymph node micrometastatic tumors and macrometastatic tumors of lung adenocarcinoma. J. Cancer Res. Clin. Oncol..

[29] Zhang J., Fujimoto J., Zhang J., Wedge D.C., Song X., Zhang J., Seth S., Chow C.W., Cao Y., Gumbs C. (2014). Intratumor heterogeneity in localized lung adenocarcinomas delineated by multiregion sequencing. Science.

[30] Liu Y., Zhang J., Li L., Yin G., Zhang J., Zheng S., Cheung H., Wu N., Lu N., Mao X. (2016). Genomic heterogeneity of multiple synchronous lung cancer. Nat. Commun..

[31] Liu Z., Liang H., Lin J., Cai X., Pan Z., Liu J., Xie X., Li C., Cheng B., Zhao Y. (2019). The incidence of lymph node metastasis in patients with different oncogenic driver mutations among T1 non-small-cell lung cancer. Lung Cancer.

[32] Nagasaka M., Gadgeel S.M. (2018). Role of chemotherapy and targeted therapy in early-stage non-small cell lung cancer. Expert Rev. Anticancer Ther..

[33] Kris M.G., Gaspar L.E., Chaft J.E., Kennedy E.B., Azzoli C.G., Ellis P.M., Lin S.H., Pass H.I., Seth R., Shepherd F.A. (2017). Adjuvant Systemic Therapy and Adjuvant Radiation Therapy for Stage I to IIIA Completely Resected Non-Small-Cell Lung Cancers: American Society of Clinical Oncology/Cancer Care Ontario Clinical Practice Guideline Update. J. Clin. Oncol..

[34] Osmani L., Askin F., Gabrielson E., Li Q.K. (2018). Current WHO guidelines and the critical role of immunohistochemical markers in the subclassification of non-small cell lung carcinoma (NSCLC): Moving from targeted therapy to immunotherapy. Semin Cancer Biol..

[35] Vansteenkiste J., Wauters E., Reymen B., Ackermann C.J., Peters S., De Ruysscher D. (2019). Current status of immune checkpoint inhibition in early-stage NSCLC. Ann. Oncol.

[36] Giuliano A.E., Connolly J.L., Edge S.B., Mittendorf E.A., Rugo H.S., Solin L.J., Weaver D.L., Winchester D.J., Hortobagyi G.N. (2017). Breast Cancer-Major changes in the American Joint Committee on Cancer eighth edition cancer staging manual. CA Cancer J. Clin..

[37] Reed J., Rosman M., Verbanac K.M., Mannie A., Cheng Z., Tafra L. (2009). Prognostic implications of isolated tumor cells and micrometastases in sentinel nodes of patients with invasive breast cancer: 10-year analysis of patients enrolled in the prospective East Carolina University/Anne Arundel Medical Center Sentinel Node Multicenter Study. J. Am. Coll Surg..

[38] Giuliano A.E., Hawes D., Ballman K.V., Whitworth P.W., Blumencranz P.W., Reintgen D.S., Morrow M., Leitch A.M., Hunt K.K., McCall L.M. (2011). Association of occult metastases in sentinel lymph nodes and bone marrow with survival among women with early-stage invasive breast cancer. JAMA.

[39] Abbosh C., Birkbak N.J., Wilson G.A., Jamal-Hanjani M., Constantin T., Salari R., Le Quesne J., Moore D.A., Veeriah S., Rosenthal R. (2017). Phylogenetic ctDNA analysis depicts early-stage lung cancer evolution. Nature.

[40] Poulet G., Massias J., Taly V. (2019). Liquid Biopsy: General Concepts. Acta Cytol..

[41] Li Y.S., Jiang B.Y., Yang J.J., Zhang X.C., Zhang Z., Ye J.Y., Zhong W.Z., Tu H.Y., Chen H.J., Wang Z. (2018). Unique genetic profiles from cerebrospinal fluid cell-free DNA in leptomeningeal metastases of EGFR-mutant non-small-cell lung cancer: A new medium of liquid biopsy. Ann. Oncol..

[42] Cassetta L., Pollard J.W. (2018). Targeting macrophages: Therapeutic approaches in cancer. Nat. Rev. Drug Discov..

[43] Noy R., Pollard J.W. (2014). Tumor-associated macrophages: From mechanisms to therapy. Immunity.

