# Supplementary material for: Oncogenic Alterations in Histologically Negative Lymph Nodes Are Associated with Prognosis of Patients with Stage I Lung Adenocarcinoma
Source: Cancers (Basel). 2022 Feb 6;14(3):824. doi: 10.3390/cancers14030824 (PMC8834139; doi:10.3390/cancers14030824)

Supplementary Materials:

Supplementary Figure S1 The expression of AE1/AE3 and BerEP4 in primary cancer tissue and corresponding lymph node with molecular alteration. Primary cancer tissue (HE, A), showed strong positive staining for AE1/AE3 (B) and Ber-EP4 (C). Lymph node with molecular alteration (HE, D) showed negative staining for AE1/AE3 (E) and Ber-EP4 (F). Original magnification were 200 times.

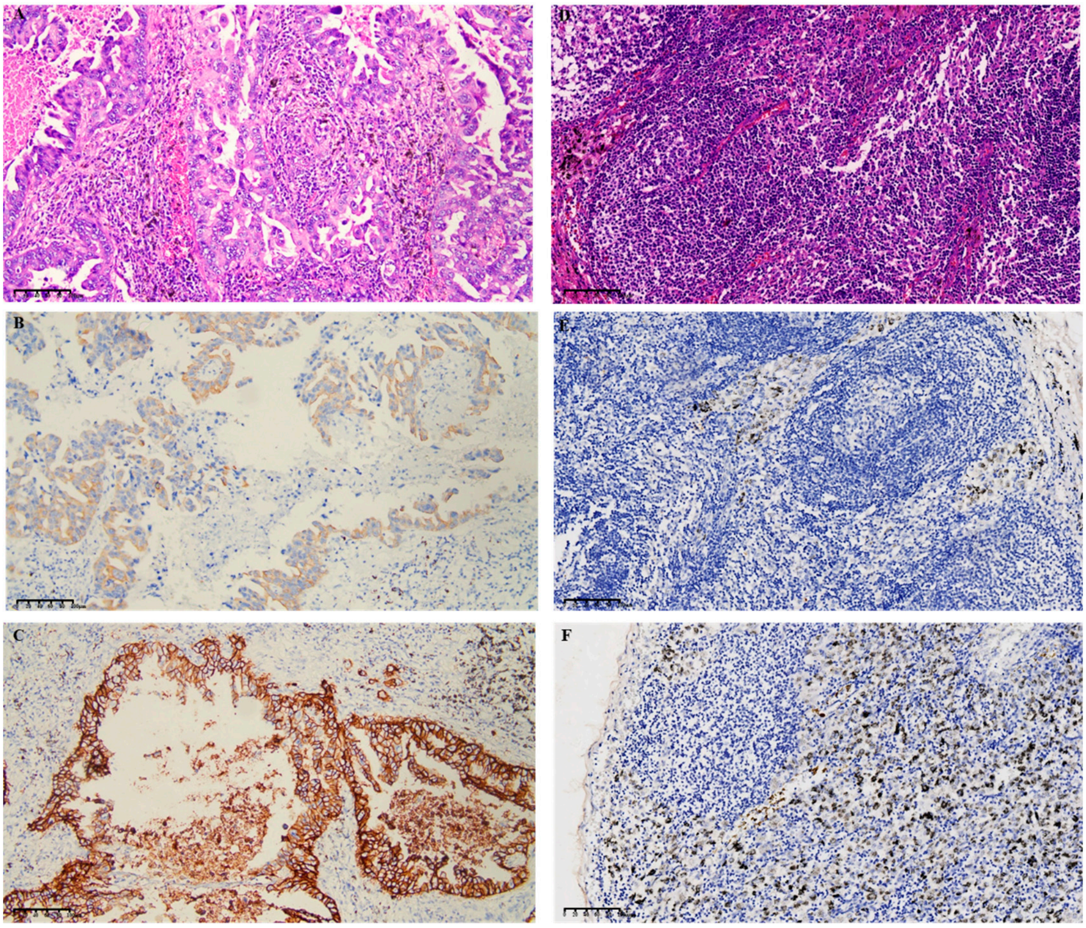

Supplement: Supplementary file 1 [file cancers-14-00824-s001.zip › cancers-1538909 Supplementary Materials.pdf]
